# Structural and Performance Optimization of Environmentally Friendly Phenolic Resin/Polyvinyl Alcohol/Pure Terephthalic Acid/Silicone Carbide (PF/PVA/PTA/SiC) Porous Composite Grinding Wheels Prepared via Freeze-Drying Methodology

**DOI:** 10.3390/polym17060758

**Published:** 2025-03-13

**Authors:** Xudong Song, Xuexue Li, Congcong Zhao, Lumin Liang, Liuwei Guo, Yuzhu Zhou, Bingqiao Zhu, Jin Peng

**Affiliations:** Henan Key Laboratory of Superhard Abrasives and Grinding Equipment, College of Materials Science and Engineering, Henan University of Technology, Zhengzhou 450001, China; 13639687728@163.com (X.L.); 18134463319@163.com (C.Z.); 18836171958@163.com (L.L.); nianjiao306@163.com (L.G.); 17828166154@163.com (Y.Z.); ykszky330@163.com (B.Z.); polymer123@163.com (J.P.)

**Keywords:** polyvinyl alcohol, pure terephthalic acid, freeze-drying, grinding wheel, pore structure

## Abstract

The traditional preparation of polyvinyl alcohol (PVA) grinding wheels typically involves hazardous chemicals such as formaldehyde and hydrochloric acid, posing significant health risks to operators and contributing to environmental pollution. In this study, we utilized the freeze-drying method to fabricate PVA grinding wheels, optimizing both the manufacturing process and the structure of the porous composite materials. The results demonstrate that phenolic resin (PF) participates in constructing a hydrogen-bonded network with PVA and pure terephthalic acid (PTA), which synergistically enhances the esterification efficiency between PTA and PVA. Furthermore, the incorporation of PTA as a crosslinking agent led to a more concentrated pore distribution, reducing the average pore size while enhancing mechanical strength. The freeze-drying duration of 42 h and 10% solid content of the PVA solution yields the favorable comprehensive porosity and mechanical performance of the grinding wheel with a unique bimodal pore structure and porosity exceeding 50%. The maximum grinding ratio was achieved at 0.81, while the surface roughness (Sa) was 0.308 μm. The freeze-drying approach significantly enhances pore uniformity and adjustability, producing grinding wheels with superior mechanical properties and performance consistency. This study presents a novel and environmentally friendly alternative to traditional PVA grinding wheel fabrication methods.

## 1. Introduction

Polyvinyl alcohol (PVA) exhibits low toxicity, biocompatibility, superior adsorption capabilities, and favorable mechanical properties, making it an ideal candidate for the development of porous composite materials [1,2,3]. Insoluble PVA can be synthesized through the acetalization of PVA with formaldehyde or butyraldehyde. The resultant polyvinyl acetal exhibits strong covalent bonding between the hydroxyl and aldehyde groups, leading to enhanced water resistance. This method is primarily employed for the production of PVA grinding wheels, which are utilized for the online grinding of cathode titanium rollers in the electrolytic deposition process of copper foil [4,5,6,7]. In the conventional process, a PVA grinding wheel is fabricated through hot pressing or chemical crosslinking techniques. The crosslinked network structure is established by blending PVA with a crosslinking agent and abrasive materials, followed by drying or hot pressing at elevated temperatures [8,9]. Due to the acetal reaction between polyvinyl alcohol (PVA) and formaldehyde under acidic conditions, the uniformity of the pores in the grinding wheel is suboptimal, resulting in the low strength of the grinding tool. Additionally, the formaldehyde molecules released during the acetal reaction pose significant environmental pollution concerns, thereby limiting its application in high-precision machining [10]. Therefore, it is imperative to investigate innovative grinding wheel preparation processes to facilitate the technological advancement and application development of PVA grinding tools.

The freeze-drying technology, also known as lyophilization or ice-templating, is a critical and versatile processing method for the design and fabrication of layered porous materials. This technique efficiently removes water from materials without compromising their internal structure by directly sublimating them at low temperatures. It finds extensive application in the development of polymer, metal, and ceramic composite materials [11,12,13,14]. The freeze-drying process involves a series of freezing and sublimation stages. Initially, the freezable water is transformed into large ice crystals. Subsequently, these ice crystals undergo sublimation, leaving behind porous structures. This results in an anisotropic, multistage pore architecture that is particularly suitable for preparing functional materials with porous structures using PVA [15].

The instability encountered during the freeze-drying of porous structures can result in structural deformation, dimensional shrinkage, and even the collapse of porous frameworks [16]. Consequently, overcoming these processing challenges and achieving precise control over porous structures are critical objectives [17]. A method combining liquid nitrogen freezing [18], freeze-drying, and hot-press molding was used to prepare a high-cellulose nanofiber (CNF)-loaded PVA nanocomposite material by Salehpour et al. [19]. Studies have shown that liquid nitrogen freezing and freeze-drying are effective approaches for improving the processability of cellulose nanofiber-reinforced polyvinyl alcohol composites, as well as enhancing their mechanical and thermal properties. Feng et al. proposed a PVA/phenolic resin (PF) composite sol-gel diamond grinding wheel, which was subjected to repeated gelation through freezing at −220 °C and subsequently sintered at 180 °C to produce the diamond grinding wheel. The findings indicated that the molecular chains of PVA and phenolic resin underwent physical crosslinking at low temperatures, thereby significantly enhancing the machining performance of the grinding wheel [20]. Yang et al. presented a novel modified phenolic resin/glass fiber composite material. The experimental results indicate that the impact strength of this modified PVA/PF composite reaches 53.21 KJ/m^2^, which is double that of the single-component materials [21]. Wan et al. synthesized a graphene-cellulose hybrid aerogel via the freeze-drying process utilizing a regenerated mixed solvent. The resultant aerogel exhibits excellent dimensional stability and a well-defined porous structure [22]. Mínguez-García et al. crosslinked PVA by incorporating citric acid (CA). The results demonstrated that under acidic conditions, the free carboxyl groups in crosslinked CA reacted with the ketone groups in curcumin to form ester bonds. Additionally, CA served as an acid catalyst, facilitating the binding of curcumin to the crosslinked PVA nanofibers [23]. Feng et al. prepared a gel-type abrasive tool utilizing PVA and PF. The results indicate that when the solid content of PVA is 6 wt% and that of PF is 8 wt%, the mechanical and tribological properties of the gels are optimized. Compared to hot-pressed abrasives, gel-forming abrasives exhibit superior microstructural uniformity, hardness consistency, and friction coefficient stability [24].

Post-treatment techniques, such as freeze-drying, can effectively remove water while preserving the three-dimensional network structure, resulting in products characterized by high porosity and a spongy appearance [25,26]. Low-temperature gelation is achieved via the freeze concentration process. Upon solvent freezing, a large pore system is formed, which is subsequently crosslinked and solidified to maintain a dense pore wall, thereby endowing the material with enhanced mechanical strength and elasticity [27]. Moreover, the controllability of aerogel pore structures can be realized by regulating the ice crystal growth direction; isotropic pores result from random freezing, whereas anisotropic pores are formed through directional freezing [28]. Meanwhile, a recent study has demonstrated a significant correlation between the fatigue properties of porous materials and their pore distribution [29].

This study utilizes the freeze-drying technology to fabricate PVA/PF/PTA/silicon carbide (SiC) composites. Phenolic resin and pure terephthalic acid (PTA) were introduced to provide additional crosslinking sites for polyvinyl alcohol (PVA), thereby enhancing the mechanical strength of the polymer matrix by modulating the degree of polymerization of PVA and the concentration of PTA. The formation and sublimation of ice crystals during the freezing process were meticulously controlled to optimize the uniformity of the pore structure. A homogeneous mixture of PVA/PF/PTA/SiC was pre-frozen to form a network-crosslinked structure, leading to the successful development of a unique porous architecture that significantly improves the strength and consistency of the grinding wheels. This work offers an innovative and environmentally friendly method for manufacturing PVA grinding wheels. The incorporation of PF and reactive substances containing carboxylic acid groups, instead of toxic chemicals such as formaldehyde and hydrochloric acid, significantly enhances the mechanical strength of PVA. Additionally, the freeze-drying technology allows for precise control over the pore structure of PVA, resulting in favorable grinding performance in advanced manufacturing.

## 2. Materials and Methods

### 2.1. Materials

Polyvinyl alcohol-1799, Polyvinyl alcohol-2699 solid particles, and pure terephthalic acid (PTA) were obtained from Shanghai Macklin Biochemical Technology Co., Ltd. (Shanghai China). Phenolic resin (PF 2461) was sourced from Jinan Shengquan Group Share-Holding Co., Ltd. (Jinan, China). Silicon carbide (SiC) was acquired from Zhengzhou Haixu Abrasive Co., Ltd (Zhengzhou, China). The particle size of SiC is W40, of which the D50 is 40 μm. Titanium alloy (TA1) (Ti > 99.5%) was purchased from ZS Titanium Co., Ltd. (Liaoning, China). Deionized water is produced through a laboratory-grade pure water system.

PVA requires sufficient water during preparation to ensure complete dissolution. Therefore, for the PVA solution system, variations in the PVA solid content directly affect the water content and viscosity of the system, which, in turn, influences the number and distribution of ice crystals, ultimately affecting the freeze-drying outcome. Excessive water leads to a reduction in the PVA solid content, making it difficult to form the system. Conversely, insufficient water results in high viscosity, which can affect the uniform dispersion of abrasives and the formation of ice crystals. Therefore, in this study, the PVA solid content in the PVA solution is controlled between 8% and 12% to ensure optimal formation and performance.

### 2.2. Green Manufacturing of the PF/PVA/PTA/SiC Grinding Wheel

**Figure 1:**
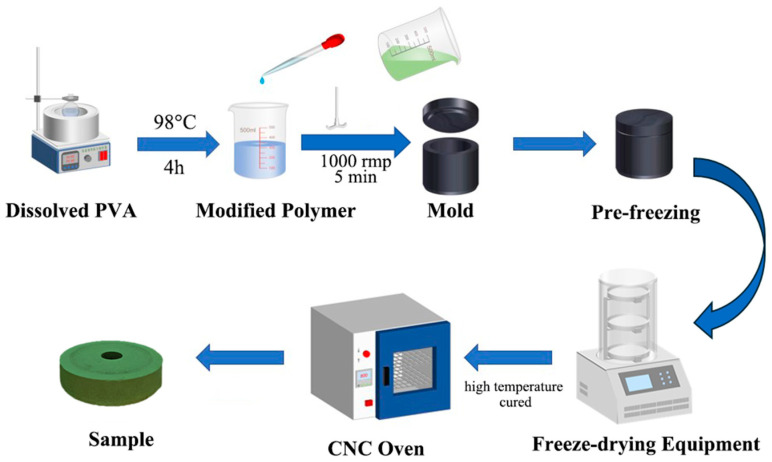
The procedure for sample preparation of PF/PVA/PTA/SiC composites.

In the preparation procedure, we eliminated the toxic crosslinking agents formaldehyde and hydrochloric acid, promoting a safer process for workers, reducing environmental impact, and reducing chemical waste. This highlights the potential for further sustainability improvements since traditional methods use formaldehyde-based crosslinking, which generates hazardous byproducts. Figure 1 demonstrates the preparation process for the test samples, which is identical to that of the grinding wheels. Firstly, polyvinyl alcohol (PVA) solid particles and distilled water are placed in a beaker, which is then heated in a thermostatic water bath at 98 °C for 2 h. The beaker is sealed with aluminum foil to retain heat and allow the PVA to dissolve gradually. Afterward, the mixture was stirred at 500 rpm until the PVA was completely dissolved in a beaker. The solution is then sealed and cooled to room temperature to prepare a (8~12)% (by mass) PVA solution for later use. If the sample requires the addition of pure terephthalic acid (PTA), the PTA is mixed with the PVA solution during the heating process at 98 °C. Next, the PVA solution, PF, PTA, and green silicon carbide abrasives are combined in a beaker and stirred with a high-power (2000 W) stirrer at 1000 rpm for 5 min. The mixture is then poured into a 304 stainless-steel mold, sealed, and placed in an insulated container. Subsequently, the mold was placed in an insulated foam box with liquid nitrogen for 2 h to perform the pre-freeze procedure. The frozen sample is transferred to a freeze-drying machine. Once the drying process is complete, the sample is placed in a forced-air oven for further processing by heating at 90 °C for 6 h, followed by 100 °C for 6 h, 110 °C for 6 h, and finally 125 °C for 6 h. After cooling to room temperature, the final test sample is obtained.

### 2.3. Measurement and Characterization

#### 2.3.1. Thermal Behavior Analysis

The analysis of the inter-component thermal behavior of the PF/PVA/PTA composite system was examined by Different Scanning Calorimetry (DSC, Netzsch 200F3, Stuttgart, Germany). The analysis was conducted under the following test conditions: the heating rate is 10 °C per minute, with a temperature range from room temperature to 275 °C, and the protective atmosphere is nitrogen. Additionally, the following sample conditions were adopted: PF is a liquid resin, while PVA and PTA are both solid particles.

#### 2.3.2. Fourier Infrared Spectral Analysis

To investigate the reaction between PVA, PF, and PTA, Fourier Transform Infrared Spectroscopy (FT-IR) was conducted using Nicolet^TM^iS^TM^ 50 (Mountain, WI, USA) with 32 scans/second. The FT-IR was recorded in the range of 400–4000 cm^−1^. For the single component test, PF is a liquid resin, while PVA and PTA are both solid particles. For the multi-component detection of PVA in a solution state, the sample of the multi-component was cured under 125 °C before the test.

#### 2.3.3. Porosity Detection

In accordance with Archimedes’ principle, the dry sample mass (M_1_) was measured using an electronic balance. The sample was subsequently boiled in distilled water for two hours. Following this, the sample was removed, and its mass (M_2_) while saturated and submerged in water, was determined using an electronic solid density meter. Additionally, the mass (M_3_) of the saturated sample in air was measured. The porosity of the material is calculated using the following formula:

(1) $$p_{t}=\frac{M_{3}-M_{2}}{M_{3}-M_{1}}\times 100\%$$

#### 2.3.4. Depth-of-Field Microscope

The morphology and the profile parameters of the PVA/PF/PTA/SiC composites were characterized and calculated by utilizing a Digital Microscope (Keyence VHX-6000, Osaka, Japan). The comparison of the parameters before and after the cure process and wear test is recorded.

#### 2.3.5. Scanning Electron Microscope

The morphologies of the fracture surfaces of the PVA/PF/PTA/SiC composites were observed. The samples were sputter-coated with gold prior to observation and investigated by the Scanning Electron Microscope (SEM, FEI Inspect F50, Hillsboro, OR, USA) at the operating voltage of 20 kV.

#### 2.3.6. Mechanical Properties

The mechanical properties of the composites were tested at room temperature using a WDW-50 electronic universal testing machine. The impact strength test was conducted according to GB/T 33835-2017 [30] and the bending strength test was carried out according to GB/T 9341-2008 [31]. The hardness was evaluated using a LX-C hardness tester according to GB/T 2411-2008 [32], which is an important indicator of the ability of the material surface to resist mechanical pressure.

#### 2.3.7. Freeze-Drying Process

The samples for the mechanical test and grinding wheels for the grinding test were prepared by a common manifold-type laboratory freeze dryer (SCIENTZ-10N/C, Ningbo Scientz Biotechnology Co., Ltd., Ningbo, China). The conventional no-load parameter temperature is −56 °C, and the freeze-drying time was controlled from 24 h to 48 h.

#### 2.3.8. Grinding Test

The MA6025 tool grinder produced by the Xianyang Machine Tool Factory (Xianyang, China) was used for the grinding test of the titanium alloy TA1 bar with four kinds of PVA grinding wheels with a diameter of 100 μm as shown in Table 1. The grinding machine parameters were as follows: spindle speed of grinding wheel is 2700 rpm; vertical feed speed of grinding wheel is 0.2 mm/min; the grinding fluid is water.

## 3. Results and Discussion

### 3.1. Thermal Behavior Analysis and Fourier Transform Infrared Spectroscopy (FT-IR) of Phenolic Resin/Polyvinyl Alcohol/Pure Terephthalic Acid (PF/PVA/PTA) Composites

**Figure 2:**
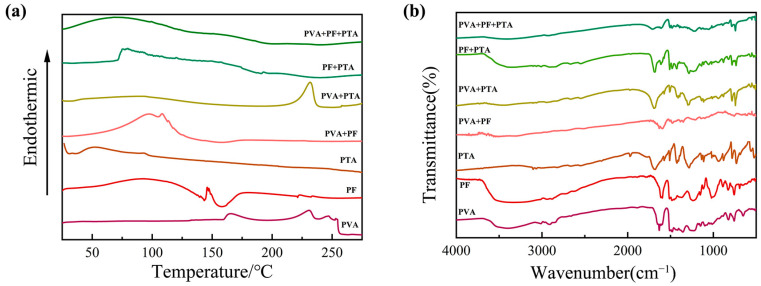
Thermal behavior analysis and Fourier Transform Infrared Spectroscopy (FT-IR) for composite systems of PF/PVA/PTA: (**a**) DSC; (**b**) FT-IR.

Firstly, Figure 2a shows the thermal behavior analysis of mono-, bi-, and ternary composite systems of PF/PVA/PTA. For the PVA single-component system, the curve exhibits a broad exothermic peak at 220–250 °C, attributed to the partial oxidative decomposition of PVA chains under a nitrogen atmosphere. PF (liquid resin) displays a broad endothermic peak near 100 °C (solvent evaporation) followed by an exothermic curing peak at 125–175 °C, corresponding to phenolic hydroxyl condensation. There are no structural phase transitions observed below 300 °C in PTA, which is consistent with its high melting point (>300 °C).

For the binary systems, a broad endothermic peak at 100–140 °C in PVA/PF was dominated by solvent evaporation (endothermic), overlapping with PF crosslinking exotherms and forming a thermal valley near 150 °C. Physical blending restricts the PVA chain mobility due to the rigid aromatic structure of PF, but no covalent interactions occur (no new FT-IR peaks in Figure 2b). A distinct exothermic peak (200–230 °C) emerges (absent in pure components) in PVA/PTA, signifying esterification between PVA hydroxyls (-OH) and PTA carboxyl (-COOH) to form ester linkages (-COO-) [33]. The FT-IR in Figure 2b validates C-O-C stretching at 1150 cm^−^^1^ and confirms ester bond formation. The curing exotherm shifts to lower temperatures (150–180 °C) with increased enthalpy in PF/PTA, indicating that PTA acts as an acidic catalyst for PF crosslinking. FT-IR reveals redshifted C=O peaks (1690 → 1680 cm^−1^) and weakened phenolic -OH signals, suggesting carboxyl–phenolic hydrogen bonding [34]. A composite endothermic–exothermic profile (120–160 °C) arises in the ternary system (PVA/PF/PTA). Dehydration of PVA (endothermic) and crosslinking of PF (exothermic) are catalytic effects of PTA-mediated composites. Suppressed decomposition above 250 °C reflects enhanced thermal resistance due to a dual-function PTA network. The PTA can significantly improve the thermal stability of composite materials through dual action (crosslinking and catalysis) in the ternary system, which provides a theoretical basis for the design of high-performance resin-based materials.

The chemical bond changes in each component before and after curing at 125 °C were characterized using FT-IR in Figure 2b. The characteristic functional groups of the three single components include the broad stretching vibration peak of the hydroxyl group (-OH) in PVA at 3300 cm^−1^, the C-O stretching vibration at 1090 cm^−1^ in the PVA backbone, and the C-H stretching vibration (-CH_2_) at 2900 cm^−1^. For PTA, the characteristic peaks include the broad O-H stretching vibration of the carboxylic acid in the range of 2500–3300 cm^−1^, and the strong C=O stretching vibration at 1690 cm^−1^. In PF, the characteristic peaks are the C=C aromatic ring skeletal vibration at 1608 cm^−1^, and the bending vibration of the phenolic hydroxyl group (-OH) at 1250 cm^−1^ [21].

In the binary system, the PVA/PF reactant spectrum shows a decrease in the intensity of the -OH peak at 3300 cm^−1^, indicating the formation of hydrogen bonds between the phenolic hydroxyl group of PF and the hydroxyl group of PVA. No changes are observed in the C-O peak at 1090 cm^−1^, suggesting that no esterification reaction occurs. In contrast, in the PVA/PTA system, the -OH peak at 3300 cm^−1^ significantly weakens, indicating the involvement of the hydroxyl groups in PVA in the reaction. The intensity of the C=O peak of PTA at 1690 cm^−1^ decreases, and a C-O-C stretching vibration peak of the ester bond appears at 1150 cm^−1^, confirming that esterification between PTA and PVA occurs at 125 °C under solution mixing conditions. In the PF/PTA reactant spectrum, the C=O peak of PTA shifts to a lower wavenumber at 1680 cm^−1^, indicating the protonation of the carboxyl group. The phenolic hydroxyl peak at 1250 cm^−1^ weakens, suggesting that the carboxyl group of PTA and the phenolic hydroxyl group of PF may form hydrogen bonds [35]. In Figure 2a, the exothermic peak in the DSC of PF/PTA shifts to a lower temperature region, indicating that PTA acts as an acidic catalyst, promoting the polycondensation crosslinking of PF phenolic hydroxyl groups. However, the infrared results confirm that no esterification reaction occurred between PF and PTA [23].

In the PVA/PF/PTA ternary system, the -OH peak at 3300 cm^−1^ further weakens, indicating that both the phenolic hydroxyl group of PF and the hydroxyl group of PVA are involved in the reaction. The intensity of the C-O-C peak at 1150 cm^−1^ is higher than in the PVA/PTA system, suggesting that PF facilitates the esterification reaction. The C=O peak of PTA shifts more significantly to 1680 cm^−1^, indicating that the acidic environment created by PF enhances the reactivity of PTA, promotes esterification, and forms a more compact crosslinked network. The intensity of the C-O-C peak in the ternary system is notably higher than in the binary system, confirming that PF significantly increases the degree of esterification. The DSC results shown in Figure 2a reveal that the exothermic peak area of the ternary system increases in the 180–220 °C range, indicating a higher extent of reaction.

The esterification reaction proceeds via an acid-catalyzed mechanism, where reaction kinetics are governed by temperature-dependent proton mobility and molecular chain dynamics. At lower temperatures, limited proton transfer and restricted chain mobility result in suboptimal reaction rates. Elevated temperatures enhance both proton migration (via thermal activation) and the segmental motion of polymer chains, synergistically accelerating the esterification efficiency. In ternary PVA/PF/PTA systems, phenolic hydroxyl groups (-OH) from PF act as acid donors, protonating the PTA carboxylic acid groups (-COOH → -COO^−^H^+^). This protonation markedly increases the electrophilicity of the PTA carbonyl carbon, facilitating a nucleophilic attack by PVA hydroxyl groups (-OH) to form ester linkages (-COO-). Concurrently, PF participates in constructing a hydrogen-bonded network with PVA and PTA, reducing the reaction activation energy through transition-state stabilization [36].

**Figure 3:**
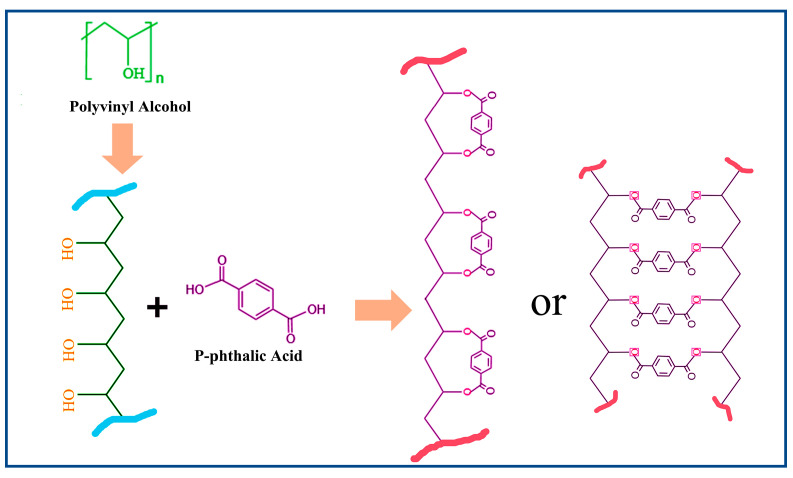
Reaction between the PVA and PTA.

Furthermore, the rigid aromatic backbone of PF imposes spatial confinement effects. The restricted chain mobility of PVA increases the local reactant concentration. The acid catalysis and steric assistance mechanisms collectively enhance the esterification kinetics in ternary systems. DSC and FT-IR data corroborate this synergistic effect, showing intensified exothermic peaks (180–220 °C) and amplified C-O-C absorption (1150 cm^−1^), respectively. This work elucidates the green composite design; the schematic diagram in Figure 3 elucidates the esterification reaction and the structural transformation between composite systems of PF/PVA/PTA, demonstrating how hierarchical interactions (acid-base, hydrogen bonding, steric effects) can be engineered to optimize reaction pathways in multi-component polymer systems [37].

### 3.2. Effect of Freeze-Drying Time on the Forming of Phenolic Resin/Polyvinyl Alcohol/Pure Terephthalic Acid (PF/PVA/PTA) Composites

**Figure 4:**
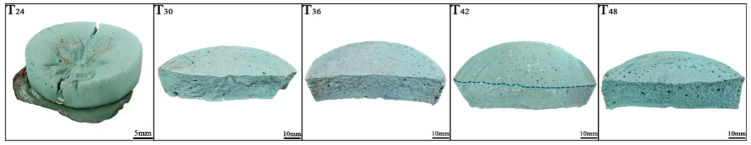
Samples under freeze-drying times of 24 h, 30 h, 36 h, 42 h, and 48 h.

The samples shown in Figure 4 demonstrate the effect of freeze-drying time on the structural integrity and morphology of grinding wheels; the samples exhibit varying degrees of deformation or structural stability after thermal curing. T24 and T30 seem to show significant deformation or cracks, indicating insufficient structural rigidity during thermal curing. Incomplete freeze-drying may leave residual moisture in the composite, causing issues such as uneven curing, cracks, or bubble formation during thermal treatment. As the freeze-drying time increases to 42 h and 48 h, the samples show improved structural uniformity and reduced cracking, suggesting a stronger internal network. Freeze-drying introduces a porous structure due to the sublimation of water; the samples presented a denser and uniform cross-sectional texture. Increasing the freeze-drying time seems to have diminishing returns in terms of improving structural integrity beyond a certain point [38]. Incomplete freeze-drying will cause incomplete removal of secondary residual water in the composite, resulting in defects in the thermal curing process, which will affect the internal network and bonding properties of the adhesive, resulting in changes in porosity. Based on the image, 42 h is likely the optimal freeze-drying time, ensuring sufficient moisture removal while maintaining porosity and structural stability [39].

During the freezing process, the water content within the resin matrix forms ice crystals that serve as templates for porosity. The pre-freezing stage, conducted at −196 °C using liquid nitrogen, induces transient nucleation, resulting in the formation of nanoscale ice crystal nuclei [18]. Rapid freezing yields smaller and more uniformly distributed ice crystals, whereas slower freezing produces larger and more irregular pores. Under vacuum conditions, ice crystals sublimate directly, thereby preventing the collapse associated with the liquid phase [25]. During the freeze-drying stage, ice crystals grow directionally along the temperature gradient, forming columnar macropores.

### 3.3. Effect of Freeze-Drying Time on the Mechanical Properties of Phenolic Resin/Polyvinyl Alcohol/Pure Terephthalic Acid (PF/PVA/PTA) Composites

**Figure 5:**
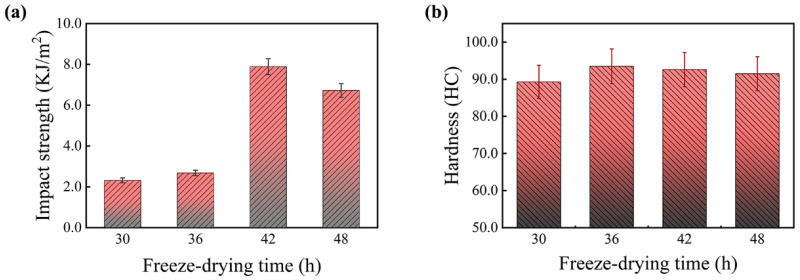
Effect of freeze-drying time on the mechanical properties of PF/PVA/PTA composites. (**a**) Impact strength; (**b**) Hardness.

Figure 5 shows the effect of freeze-drying time on the mechanical properties of PF/PVA/PTA composites, where all samples were subjected to thermal curing at 125 °C. The freeze-drying time has a significant impact on the impact strength. As the freeze-drying time increased from 30 h to 42 h, the impact strength improved from 2.32 KJ/m^2^ (T30) to its peak value of 7.89 KJ/m^2^ (T42), representing a 340% increase. However, further extending the freeze-drying time to 48 h caused the impact strength to decrease slightly to 7 KJ/m^2^. It is likely attributable to stress concentration resulting from the excessive growth of ice crystals. This observation aligns with the findings in cellulose nanofiber-reinforced PVA composites, highlighting the necessity to achieve a balance between drying efficiency and structural integrity [19]. When the freeze-drying time is very short, residual moisture remains in the composite, preventing the formation of an ideal porous structure. As a result, the cured material lacks structural density, making it less capable of effectively dispersing external forces, leading to lower impact strength. With prolonged freeze-drying, the internal moisture is fully removed, resulting in a uniform and stable microporous structure. This structure enhances the toughness and energy absorption capacity of the material after thermal curing, significantly improving its impact strength. However, excessive freeze-drying can lead to the formation of overly developed pores or micro-cracks during the drying process, which compromises the structural integrity of the material, causing a slight decrease in impact strength.

The material’s hardness exhibits minimal fluctuation under varying freeze-drying durations. Primarily, the hardness is influenced by heat curing and is strongly associated with the matrix components and the reaction extent of the PF/PVA/PTA composites, followed by the density of the microporous structure. PVA possesses inherent flexibility, and the incorporation of PTA facilitates the crosslinking of PVA, thereby enhancing the material’s hardness. PF consists of rigid benzene ring groups, which limit the molecular chain’s torsional space, resulting in a highly rigid composite material with reduced flexibility and increased brittleness [24]. The crosslinking reaction of PF/PVA/PTA composites during thermal curing at 125 °C yields a stable structure. Hardness reflects the overall structural properties of the material rather than localized characteristics. Although different freeze-drying times may cause minor alterations in the pore structure, their impact on hardness remains minimal. The appropriate freeze-drying duration can effectively eliminate excess moisture and establish a stable porous structure, which serves as an essential precondition for the subsequent thermal curing of the material [28]. During the thermal curing process, the matrix properties are significantly enhanced, the crosslinking reaction of the resin is facilitated, leading to matrix solidification, and the hardness and structural integrity of the material are markedly improved. A freeze-drying period of 42 h combined with a thermal curing temperature of 125 °C can effectively enhance the overall mechanical performance of PF/PVA/PTA composites.

### 3.4. Comprehensive Mechanical Properties Analysis of Phenolic Resin/Polyvinyl Alcohol/Pure Terephthalic Acid/Silicone Carbide (PF/PVA/PTA/SiC) Composites

**Figure 6:**
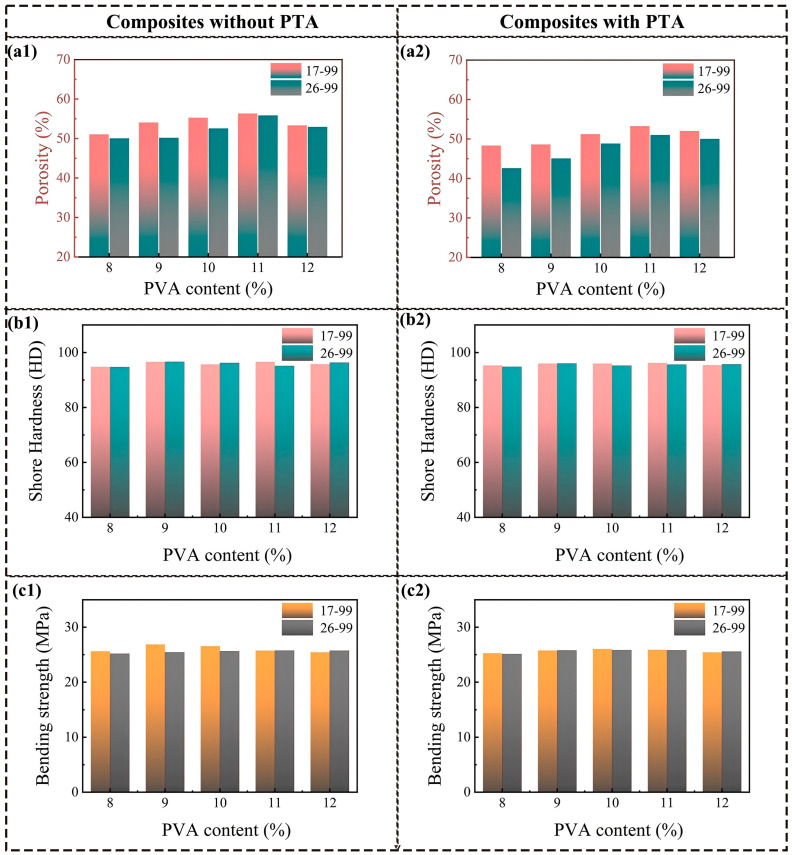
Comprehensive mechanical properties of PF/PVA/PTA/SiC composites: (**a1**,**a2**) Porosity; (**b1**,**b2**) Shore hardness; (**c1**,**c2**) Bending strength.

Figure 6 illustrates the impact of PVA solid content on the porosity and mechanical properties of the PVA/PF/PTA/SiC composite system. As the PVA solid content increases from 8% to 12%, the water content in the composite slurry decreases, and the viscosity of the slurry increases. The higher viscosity thickens the solution, thereby restricting the mobility of water within the solution. This high viscosity inhibits the rapid diffusion of water, aiding the formation of smaller ice crystals, which, in turn, leads to a smaller and more uniform pore structure after the freeze-drying procedure. As a result, regardless of whether PTA is added, the porosity initially increases and then decreases. When the PVA solid content reaches 11 wt%, the porosity of the composite system reaches its maximum value, with values of 56.34% (PVA-1799), 55.85% (PVA-1799), 53.26% (PVA-1799/PTA), and 51.01% (PVA-1799/PTA). At the PVA content of 11%, the water content and viscosity of the system are in an optimal balance, allowing for the formation of sufficiently small ice crystals while maintaining a reasonable pore distribution, resulting in the maximum porosity. Moreover, this balance enhances the stability of the system, suppressing excessive shrinkage of the pore structure and ensuring a more stable porosity. Further increasing the PVA content leads to a reduction in water content and further shrinking of the ice crystals. Excessively high viscosity, however, results in an uneven pore structure, which reduces porosity.

PVA-1799 was a kind of lower degree of polymerization and more loosely packed molecular chains, making it easier to form a loose network structure within the system. During the freeze-drying process, the loose structure promotes the formation of ice crystals, resulting in a better porosity. When PTA is incorporated, the viscosity of the composite system increases, and crosslinking occurs between the PTA and PVA molecules, further enhancing the structural stability of the system. This compresses the growth space for ice crystals, leading to a reduction in the composite material’s porosity. At this point, the higher polymerization degree of PVA-2699 and the enhanced crosslinking effect result in lower porosity in the composite material. The matrix of the composite system consists of PF, PVA, and PTA. The mechanical performance remains stable due to the lack of significant changes in the content of main components. For resin-based porous composites with similar porosity, the composition of the resin matrix has a more significant impact on mechanical performance. In the composite system without PTA, the overall mechanical performance exhibits an inverse relationship with porosity. As porosity increases, the supporting wall structure of the pores decreases, leading to a slight decline in mechanical performance. In contrast, in the composite system containing PTA, the crosslinked structures generated by esterification reactions can effectively reduce the impact of PVA polymerization degree on the performance of the composite material. Therefore, when the PVA content is 10%, a moderate porosity (50–56%) can be achieved, allowing the material to possess both high impact strength and hardness.

### 3.5. Pore Structure and Distribution of Polyvinyl Alcohol/Phenolic Resin/Pure Terephthalic Acid/Silicone Carbide (PVA/PF/PTA/SiC) Composite Grinding Wheel

**Figure 7:**
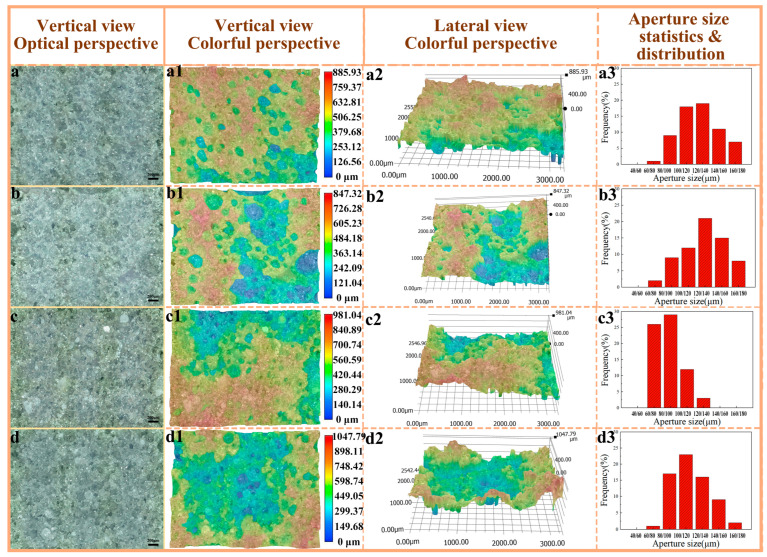
Color depth-of-field photographs of pore structure and statistics of pore size distribution. (**a**–**a3**) PF/PVA-1799; (**b**–**b3**) PF/PVA-2699; (**c**–**c3**) PF/PVA-1799 + PTA; (**d**–**d3**) PF/PVA-2699 + PTA.

The samples, obtained from the natural fracture surface following impact performance testing, were examined at both horizontal and oblique angles using a super-depth microscope in Figure 7. In Figure 7a, the fracture surface exhibits a relatively smooth texture, with evenly distributed pore sizes and low pore connectivity, showing no signs of collapse or aggregation. The presence of large, dark pore regions is minimal, suggesting a predominance of closed pores over open ones. Statistical analysis reveals that the proportion of pores within the 100–120 μm range is 17.8%, while those in the 120–140 μm range account for 19.2%. In contrast, the fracture surface depicted in Figure 6(b1,b2) is notably rougher, characterized by a greater abundance of large pores and extensive pore collapse and accumulation as observed in the side view. The pore size distribution remains relatively uniform, with 14.3% of pores falling within the 100–120 μm range and 21.7% in the 120–140 μm range. The dark areas have expanded, indicating an increase in both the number and size of pores. This phenomenon can be attributed to the higher polymerization degree of PVA-2699 compared to PVA-1799, where the elevated molecular weight hinders the uniform distribution of ice crystals during pore formation, leading to significant variations in pore location and size. Consequently, the pore depth and surface roughness are observed to increase with the rise in PVA molecular weight [40].

The fracture surface of the sample incorporating pure terephthalic acid is depicted in Figure 7c, exhibiting a notable increase in the formation of relatively flat and small pores. The dimensions of the fusion holes have expanded, and the aperture distribution has become more uneven yet more concentrated. Specifically, 60–80 μm apertures account for 25.9%, while 80–100 μm apertures represent 29.3%. The surface morphology has become more intricate, with a significant rise in both the depth and number of pores. Upon the addition of pure terephthalic acid, the darkened regions in Figure 7(c2) have expanded, suggesting an increase in the number of openings and a concentration of larger pore sizes, albeit with a reduction in overall size. In Figure 7d, the fracture surface exhibits greater roughness, characterized by extensive areas of porosity-induced defects, including large holes and pronounced depth variations. The number of open pores has increased, and the porosity structure has become interconnected. However, a comparison with the pore size and distribution in Figure 6(b1,b2) reveals a decrease in pore size and a more concentrated distribution, with 80–100 μm, 100–120 μm, and 120–140 μm apertures accounting for 17.2%, 23.2%, and 16.1%, respectively.

The incorporation of pure terephthalic acid facilitates additional crosslinking reactions, thereby enhancing the porosity of the network structure. However, this also leads to a reduction in material compatibility and the formation of larger pores. As a result, the enlargement of porosity intensifies stress concentration, potentially diminishing the material’s impact resistance. Conversely, the presence of larger pores may improve the material’s energy absorption capacity, making it suitable for applications in energy absorption or cushioning [41].

### 3.6. Scanning Electrone Microscope (SEM) of Pore Structure and Abrasive Particle Distribution of the Polyvinyl Alcohol (PVA) Grinding Wheels

**Figure 8:**
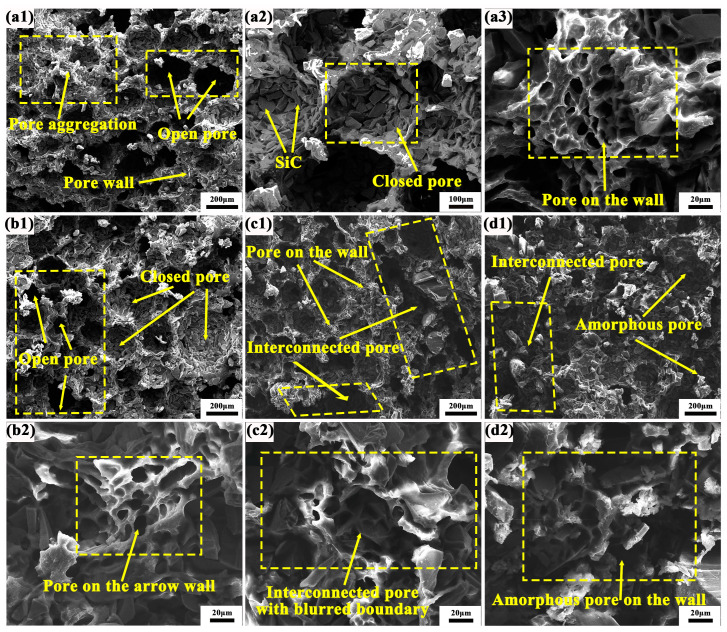
Microstructure of PVA/PF/PTA/SiC composite grinding wheel: (**a1**–**a3**) PF/PVA-1799; (**b1**,**b2**) PF/PVA-2699; (**c1**,**c2**) PF/PVA-1799 + PTA; (**d1**,**d2**) PF/PVA-2699 + PTA.

Figure 8 presents the SEM fracture morphology of grinding wheels incorporating various composite material components. For the sample in Figure 8(a1), the pore profile of the composite material is distinctly visible against the backdrop of the macroscopic ultra-depth-of-field microstructure. Clear boundaries and pore walls are present, alongside uniformly distributed closed and open pores. Notably, the open pores exhibit a longitudinally penetrating porosity structure, with numerous instances of porous wall accumulation. Upon increasing the magnification to Figure 8(a2), it becomes evident that the pore structure comprises a substantial amount of SiC abrasives, devoid of excessive resin binder as a coating. This configuration facilitates the continuous grinding capability of the grinding wheels and the self-sharpening property of the abrasives, thereby maintaining tool sharpness. As illustrated in Figure 8(a3), at higher magnification, the pore wall reveals approximately 10 μm-sized micropores formed by the resin binder on the rhomboid pore wall. These micropores exist independently, exhibiting uniform shape and distribution. In the PF/PVA-1799 system, the sample structure exhibits a bimodal distribution, with ice crystals dispersed throughout the system [42]. Water, acting as a solvent, also contributes to the formation of small pores within the macromolecular structure. The coexistence of large pores (60–120 μm) and micropores (10–20 μm) enhances the heat dissipation capacity of the grinding wheel and improves its self-sharpening properties [43].

When PTA is introduced into the PF/PVA-1799 system, as illustrated in Figure 8(b1), the size of the open pores diminishes and they are distributed in various forms at the interface, while the size of the closed pores formed tends to increase. The enlarged closed pores can retain a greater amount of SiC abrasives within their walls, thereby enhancing the sharpness support for grinding. At this stage, a distinct boundary between the pore wall and the stomata remains visible, with no evidence of pore wall aggregation. In Figure 8(b2), it is evident that the thickness of the pore wall decreases and narrows, leading to an uneven distribution and size of pores on the wall. The addition of PTA facilitates the esterification of the carboxyl group and the hydroxyl group under conditions of high temperature and acidity, promoting the crosslinking of PF/PVA/PTA, which further reduces the volume of the polymer structure and compresses the pore space left by the in situ ice crystals [44].

**Figure 9:**
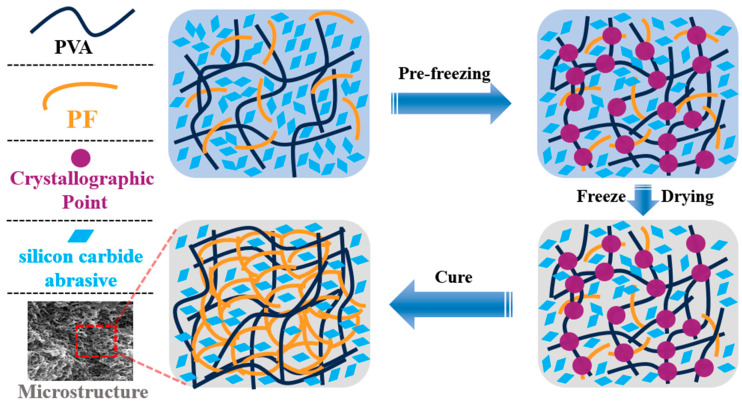
Microstructure schematic of PVA/PF/PTA/SiC composite grinding wheel.

In Figure 8(c1), it is evident that the pore size has been significantly diminished, leading to the formation of a transversely penetrating pore structure. Additionally, a distinct and fine micropore structure is discernible at the upper portion of the pore wall. These alterations result in a decrease in the number of pore walls engaged in the grinding process simultaneously, thereby reducing the quantity of SiC abrasives participating in the grinding action, and consequently impacting the grinding efficiency per unit time. In Figure 8(d2), it is observable that the stomata exhibit an irregular shape and a discontinuous state, leading to spatial disintegration. In Figure 8(a2,c2,d2), the micropores on the pore wall are interconnected with indistinct boundaries. When the PVA in the adhesive system transitions from 1799 to 2699, the molecular weight of the adhesive system increases, hindering the growth of ice crystals and resulting in a reduction in pore size. The incorporation of PTA enhances intermolecular crosslinking, further increasing the network density and limiting pore formation. This inhibits the growth of ice crystals and reduces the size of pores during the freeze-drying process (Figure 9). It also improves system compatibility, prevents phase separation, and promotes the formation of a more uniform pore structure. Furthermore, it alters solvent migration behavior and mitigates pore expansion.

### 3.7. Grinding Test for Four Kinds of Polyvinyl Alcohol (PVA) Grinding Wheels

**Figure 10:**
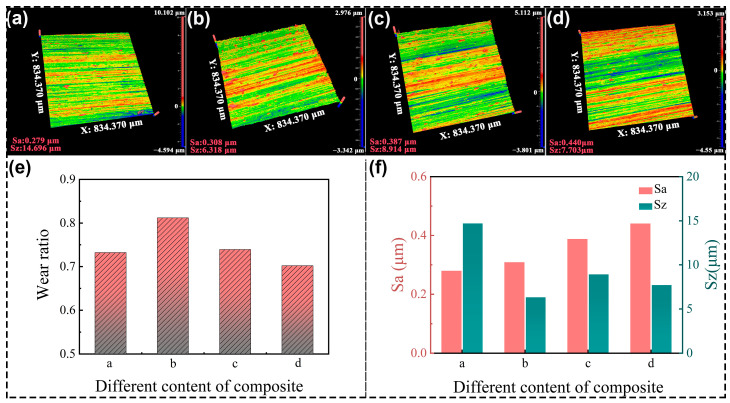
The grinding performance of PVA/PF/PTA composite wheel: (**a**–**d**) TA1 surface quality processed by different grinding wheels; (**e**) Wear ratio of the grinding wheel; (**f**) Surface roughness of the TA1.

Four grinding wheels were prepared successively based on the materials in Table 1, and the grinding results are shown in Figure 10. The lowest surface roughness parameter of Sa is 0.279 μm, which is observed in Figure 10a. The color scales predominantly feature green hues, with a notably uniform distribution, while the parameter of Sz is the highest at 14.696 μm. The grinding wheel made of PF/PVA-1799 without PTA is relatively soft to achieve a smooth surface, but disabled to perform the mass removal of the TA1 material. Figure 10b demonstrates the results processed by the grinding wheel with PTA. The Sa increased to 0.308 μm, but the Sz decreased to 6.318 μm. The scratches in Figure 10b are more pronounced compared to those in Figure 10a. Meanwhile, Figure 10e shows that the grinding wheel “b” containing the PTA with relatively hard properties exhibited the highest wear ratio of 0.81 in the grinding test. The intermolecular crosslinking attributed to the enhancement of the resin matrix and uniform distribution of pores (Figure 8(b1,b2)) are advantageous for enhancing the grinding performance of porous composite materials, ensuring an optimal distribution of SiC abrasives on the pore walls to maximize the involvement of abrasive particles in the grinding process per unit contact area.

The highest roughness parameter Sa is 0.440 μm in Figure 10d. The color scales in Figure 10c,d demonstrated that the surface scratches are particularly conspicuous and appear on the profile of TA1 compared to those in Figure 10a,b, while the parameter Sz has a lower value at 7.703 μm and 8.914 μm. By integrating the analysis of wear ratio and surface quality of TA1 data in Figure 10e,f, we can obtain the following results. The grinding wheel with PVA-2699 demonstrates the minimum wear ratio and the maximum Sa value. The high molecular weight of PVA in the system will increase the slurry viscosity, thereby affecting ice crystal formation, and subsequently impacting pore structure development. While the addition of the high molecular weight of PVA and PTA can further enhance the hardness of the system and material removal capability, the bigger size of the pores decreased the volume of the resin matrix and the continuous grinding capacity of the grinding wheels. Therefore, the grinding wheels composed of different materials can achieve varied grinding performances. In practical applications, it is possible to prepare the grinding wheels with specific parameters tailored to the requirements of particular field conditions. For grinding wheels, it is not advisable to focus solely on a single pore structure or specific performance attribute. Instead, an optimal balance between the mechanical strength and porosity of the bonding matrix must be achieved to attain the best overall performance.

## 4. Conclusions

This study successfully demonstrated a novel freeze-drying method for the preparation of PVA/PF/PTA composite grinding wheels, offering an environmentally friendly and efficient alternative to traditional methods that involve hazardous chemicals. The incorporation of pure terephthalic acid (PTA) significantly enhanced the material’s mechanical properties and pore structure, thereby facilitating additional crosslinking reactions. The freeze-drying process enabled precise control over the pore structure, resulting in a unique bimodal porosity characterized by large pores (60–140 μm) and micropores (10–20 μm). The introduction of PTA led to a more concentrated pore size distribution, which is crucial for balancing porosity and mechanical strength to enhance the grinding performance. The FT-IR analysis confirmed esterification between the hydroxyl groups of PVA and the carboxyl groups of PTA, as evidenced by the formation of new C-O-C bonds and a reduction in hydroxyl group intensity. This chemical interaction improved the structural stability and bonding matrix. Grinding tests revealed that the optimized composite structure supported effective abrasive retention and energy absorption, thereby improving the grinding efficiency while maintaining high-quality surface finishes on TA1 titanium alloy bars.

## Figures and Tables

**Table 1:** Components of PF/PVA/PTA/SiC for the grinding wheel samples. (✓indicated the incorporation of the component, “-” indicated none).

Component (Mass Content)	PF (75)	PVA-1799 (15)	PVA-2699 (15)	PTA (10)	H_2_O (135)	SiC-W40 (250)
a	✓	✓	-	-	✓	✓
b	✓	✓	-	✓	✓	✓
c	✓	-	✓	-	✓	✓
d	✓	-	✓	✓	✓	✓

## Data Availability

Data are contained within the article.
